# Construction and application of the "Internet + public health service" supply evaluation index system

**DOI:** 10.1371/journal.pone.0293341

**Published:** 2024-02-23

**Authors:** Yu Yong, Yu Ming, Chen Xia, Zeng Long

**Affiliations:** College of Public Administration and Law, Hunan Agricultural University, Changsha, Hunan, China; Sreenidhi Institute of Science and Technology, INDIA

## Abstract

To construct an index system for evaluating the supply of Internet+ public health services, and to provide a practical tool for assessing the supply of Internet+ public health services in an objective and scientific manner. The research team drafted the index system framework by combing the literature. The Delphi method was used to determine the content and weight of the index through two rounds of expert consultations. At the same time, the Cronbach coefficient and factor analysis were adopted to test the reliability and validity. What’s more, the analytic hierarchy process and the TOPSIS method were applied to analyze the empirical data of 15 counties and cities in the eastern, central, and western regions of China. The constructed indicator system includes 3 first-level indicators, 6 second-level indicators and 29 third-level indicators. Through reliability and validity tests, the stability and practicability of the index system are demonstrated in empirical research. The evaluation index system constructed in this article can be applied to the performance evaluation of Internet + public health service.

## Introduction

With the development of mobile Internet, the informatization, networking, and intelligence of public health services have become the direction of future development. It was clearly proposed to "innovate the‘Internet +’public health service" in the《the Opinions of the General Office of the State Council on promoting the development of "Internet + Medical and Health"》in 2018 [1], to realize the modernization of medical and health services, and meet the new needs of health care in the information age. In the major public health emergencies against the new pneumonia epidemic, "Internet + public health services" played a significant role [2] in ensuring the smooth implementation of the "Five Ones" services with the main objectives of "integrated" sharing, "one code" integration, "one-stop" settlement, "one network" administration, and "one chess" fight against the epidemic. In the context of the normalization of the prevention and control of COVID-19 and the building of a human health community, people’s digital health literacy has been rapidly improved, and they generally use the Internet platform to pay attention to the epidemic situation and health information. And the "Health Code", an innovative achievement of digital warfare against the epidemic, has been promoted throughout the society. Educational institutions integrated educational resources and online platforms to strengthen guidance and health interventions for students, and created an online teaching model of "no classes, no school" to effectively reduce the risk of cross-infection of COVID-19 [3]. Medical and health institutions reduced the pressure of offline treatment and disease infection rate through O2O (i.e. Online to Offline) mode such as online consultation and home delivery of medicine. At the same time, the information sharing platform was also used to strengthen the collaboration between urban and rural medical and health institutions, guide the radiation of urban high-quality medical resources to rural areas, which promote the development of medical communities and medical consortia and improve the ability of medical and health institutions in rural areas to "prevent serious illnesses" as well as effectively reducing the mortality rate of diseases. Thus, with the coupling application of information technology such as Internet of Things, big data, cloud computing and medical and health services, "Internet + public health services" became the preferred solution for people’s health protection with the focus on prevention. It can more accurately meet the needs of the public for convenient, personalized and intelligent health services, providing a technical support platform for the active health of the people, which empower them to be the "first person responsible for health", and enhance the satisfaction and sense of access to public health services. However, what cannot be ignored is that in the actual practice process, due to the differences of basic conditions, resource input, social environment and policy implementation in different regions, the construction degree of “Internet + public health service” is insufficient and unbalanced [4]. This situation has affected the modernization and equalization of public health services, which is not conducive to the advancement of the goal of "Healthy China". Therefore, it is necessary to construct a set of scientific and practical index system to analyze and evaluate the supply situation of “Internet + public health service”, so as to measure the innovation effect of “Internet + public health service” and improve the relevant service content.

## Construction of index system

### 1. Design concept

#### Analyze the resource allocation status of Internet + public health services from the perspective of infrastructure construction

The importance of health to individuals and society is self-evident. It is the most basic and important right of citizens [5], and basic public health services can provide equal health service guarantees for all residents. In order to achieve this goal, it is necessary to improve the accessibility of basic public health services through relevant institutional arrangements and technical means, so that all members of the society can easily enjoy basic public health services. The public health services supply is the most important function of the health system. Some international models on health system and the health system performance evaluation system of WHO have emphasized the status of health service supply, such as the health system model [6] proposed by WHO in the World Health Report as well as the Roemer model [7]. Both have established the corresponding mechanism based on the perspective of health service delivery. In the process of health service provision, human, financial, material, information, management and other resources all play a very important role. The Internet is the most convenient communication bridge in today’s information age, which can build an effective digital platform for the optimal allocation of resources and cannot be ignored in the development process of any field [8]. When designing the index system, it is believed that only improving the accessibility of basic public health services can ensure that every social citizen enjoy basic public health services with equal opportunity [9]. The Internet is an important way to improve the accessibility of services, which can be widely applied in public health services. However, the Internet + public health service must have a solid foundation in the aspects of public health service informatization and health service organization construction, so as to achieve the breakthrough of "1+1>2". Therefore, two indicators were selected: "Public Health Service Information Platform" and "Public Health Service Organization Construction" from the aspects of network channel construction and organizational structure construction to measure the foundation of Internet + public health services. This will not only be able to assess the status of local basic public health offline services, but also reflect its ability to expand to network services. Only by strengthening the infrastructure of Internet + public health services, can the network, information and digitization of public health service projects be smoothly promoted.

#### Relying on basic public health service items to measure its online and offline supply status

The national basic public health service project is currently the public health service content that is most closely related to the health of residents, with the most extensive influence and the largest number of beneficiaries. Thus, The optimization of its supply model has very important social significance. Meanwhile, we truly believe that it is more suitable for most projects of basic public health services to use the Internet platform to improve the services accessibility and achieve the goal of convenience and benefit for the people. In this work, the online and offline supply status of basic public health service projects was taken as the measurement index of the Internet + public health service supply process. Combining with the current implementation of basic medical and health institutions, it can be found that some service items involve communication and coordination among medical and health professional institutions, and the degree of networking is very high. The special network systems established by countries or regions was conducted to standardize management, such as the network reporting system for infectious diseases and public health emergencies. In terms of resident health services, as the service content involves the interaction and communication between the supply and demand, the degree of network promotion still needs to be strengthened. Although the network platform was used in some primary-level medical and health institutions to provide services such as appointment, consultation, follow-ups in practice, the relevant network service data was seldom collected by the primary-level medical and health institutions, owing to the fact that a systematic and standardized management system has not yet been introduced at the policy level. Therefore, in this study, the current indicators commonly used by primary care institutions are used to comprehensively assess their online and offline supply status based on the reality.

#### Measure the effectiveness of Internet + public health services by the health status and satisfaction of residents

The purpose of applying Internet information technology to basic public health services is to improve the accessibility of services, thereby improve the health status of all social citizens [10]. The effectiveness of its implementation is mainly carried out from two dimensions: health status and attitude of residents. The health effects of residents are mainly reflected in the management of chronic diseases and the prevention of infectious diseases. Of course, the people’s physical and mental health are affected by many factors, while the public health service is one of them. It is also necessary to use comprehensive indicators to measure health outcomes for specific analysis, and to consider the comparison of these measurement indicators in different regions and different populations. Residents’ attitudes mainly include the awareness rate of residents on basic public health services and the rate of residents’ satisfaction with basic public health services. The effectiveness of the implementation of basic public health services and the gap between them and the needs of residents are measured from both the supply and demand sides, so as to be continuously improved.

## 2. Research methods

### Delphi method

The Delphi method is a relatively common screening method for the indicator system, which uses multiple rounds of anonymous letter inquiries to promote consensus among experts [11]. The implementation steps in this study include: ①Establish an expert database, select appropriate experts to participate in consultations. [12,13] ②Summarize and analyze relevant literature, and design an indicator system of public health service supply evaluation and expert consultation questionnaire about Internet + public health services. ③Issue consultation questionnaires to experts and collect them in time. ④Collate and analyze the collected expert opinions. ⑤Issue and recover the second round of expert consultation questionnaires.⑥Summarize and analyze the consultation results again. According to the expert opinions, the revisions would be continuing in this cycle until the experts generally agree [14]. ⑦Finally, get the final results by comprehensively analyzing the opinions of the experts [15].

### Statistical analysis of expert consultation

(1) Expert Positive Coefficient Calculation Method

The effective recovery of expert consultation table reflects the enthusiasm of experts to participate in the study. The calculation method is, Expert consultation table recovery rate = number of experts involved / total number of experts * 100%.

(2) Expert authority Coefficient Calculation Method

The expert authority coefficient (C_r_) is determined by the judgment coefficient (C_a_, that is the expert ’s judgment basis) and the familiarity coefficient (C_S_, that is the expert ’s familiarity with the problem). The calculation formula is,

$$C_{r}=\frac{C_{a}+C_{s}}{2}.$$ (1)

The authority coefficient fluctuates between 0 and 1. The closer the value is to 1, the more authoritative the expert is.

(3) Expert coordination coefficient calculation method

The value of expert coordination coefficient w lies between 0 and 1, and the closer it is to 1, indicating that the experts reach a unanimous decision. The calculation formula is,

$$\frac{12\sum_{j=1}^{k}{R_{j}}^{2}-{3b}^{2}{k(k+1)}^{2}}{b^{2}k(k^{2}-1)}$$ (2)

Where b is the number of experts involved, k is the number of indicators, *R*_j_ is the sum of ranks assigned to the *j*_th_ indicator.

## 3. Indicator construction results

The framework of the index system was initially drafted through literature combing. At the same time the Delphi method was used to determine the content of the index and its weight through two rounds of expert consultation.

### Basic information of experts

The authority and representativeness of experts is a key factor affecting the quality of expert consultation, and the corresponding conditions in terms of professional field, title and education, work experience, etc. have been set. (1) The experts come from the fields of public health and mobile health, including universities and research institutions, health administrative departments, primary-level health service institutions and other enterprises and institution. (2) Experts have obtained intermediate and above titles, with solid theoretical foundation. (3) Experts possess a rich work experience for more than 10 years; After selection, invitations were sent to 17 experts for consultation, and finally 15 experts accepted the invitation and completed two rounds of consultation. In order to make the constructed index system more representative and more widely used, experts were invited from universities (7/15), primary health care service organizations (5/15) and health administration agencies (3/15) in Hunan, Shandong and Guangdong provinces and cities, and their work and research fields are closely related to public health services. In terms of titles and education, all of them are of intermediate level or above, including 11 senior titles, accounting for 73% (11/15) of the total number of experts in the group, and 10 experts with postgraduate degrees, accounting for 67% (10/15) of the total number of experts in the group, including 7 with doctoral degrees. In terms of working experience, 15 experts have participated in the work for an average of 18.3 years, and have accumulated rich knowledge and achieved relatively excellent achievements in the relevant fields. The basic situation of the expert group is shown in Table 1.

**Table 1:** The basic situation of the expert group.

category		Number	Percentage (%)
Working hours of experts	10–20 years	7	40
	21–30 years	5	48
	31–40 years	3	12
Educational background of experts	college degree	2	8
	bachelor degree	3	20
	master degree	3	20
	doctor degree	7	52
professional title	middle level	4	27
	high level	11	73
Distribution of Expert Job	health administration	3	20
	health service agency	5	33
	University teachers / researchers	7	47

### Expert enthusiasm

Two rounds of expert consultation were conducted in the research. In the first round of consultation, 17 experts in relevant fields were invited, and 15 effective expert consultation forms were recovered, in which the expert positive coefficient reached 88%. In the second round, a total of 15 experts were consulted, and 15 effective expert consultation forms were recovered, and the positive coefficient of experts was 100%.

### Degree of expert authority

In the process of expert consultation, the average values of the three first-level indicators—service accessibility, service implementation, and service results, reached 0.782 and 0.788 respectively. The influence of the judgment criteria on experts was moderate. The average values of the familiarity coefficients were 0.843 and 0.849 respectively, indicating that experts involved in the consultation were familiar with the contents of the consultation. The average values of the authority of experts of the three first-level indicators were 0.812 and 0.819 respectively, which means that the expert authority degree was relatively ideal. The s**tatistical table of two rounds of expert authority degree** is shown in Table 2.

**Table 2:** Statistical table of two rounds of expert authority degree.

Indicators	The first round of consultation			The second round of consultation		
	*c*_*α*_	*c*_*s*_	*c*_*r*_	*c*_*α*_	*c*_*s*_	*c*_*r*_
Internet + Public Health Service Basis	0.780	0.822	0.801	0.789	0.829	0.809
O2O supply of public health service projects	0.789	0.869	0.829	0.791	0.875	0.833
Internet + public health service results	0.776	0.837	0.807	0.783	0.842	0.812
Average	0.782	0.843	0.812	0.788	0.849	0.819

### Degree of expert coordination

The indicator system framework initially drafted in this study contained 3 first-level indexes, 6 second-level indexes and 36 third-level indexes. After the first round of consultation, the corresponding indicator content was adjusted in the indicator system of the second round of expert consultation according to expert feedback. The numbers of indicators were 3 first-level indicators, 6 second-level indicators, and 35 third-level indicators. The coordination coefficient W of the first round of expert consultation was 0.338(*χ*^2^ = 236.835, P<0.005), while that of the second round of consultation was 0.357(*χ*^2^ = 242.696, P<0.005), which the difference was statistically significant. The d**egree of coordination between two rounds of expert opinions** is shown in Table 3.

**Table 3:** Degree of coordination between two rounds of expert opinions.

	The first round of consultation	The second round of consultation
Index number	36	35
Coordination coefficient	0.338	0.357
*x* ^2^	236.835	242.696
P	<0.005	<0.005

### Screening of evaluation indicators

According to the statistical results of two round of recycling questionnaires, the boundary value method was adopted to screen the evaluation indexes on the basis of fully considering the modification opinions of experts. That is, on the basis of calculating the full score frequency, mean and standard deviation of a certain index, the evaluation grade is divided by adding and subtracting the standard deviation of the mean and the standard deviation, and the score is assigned respectively, which is used as the basis for selecting and selecting the indexes. The method of calculating the grade boundary value of full score frequency and arithmetic mean is “boundary value = mean-standard deviation”, and the index whose calculated score is higher than the boundary value is selected. The coefficient of variation grade boundary value calculation method is “boundary value = mean + standard deviation”, and the index whose calculated score is lower than the boundary value is selected [16]. The final evaluation index system includes 3 first-level indexes, 6 second-level indexes and 29 third-level indexes. The boundary value table of the two rounds of expert consultation screening indicators is shown in Table 4.

**Table 4:** Boundary value table of two rounds of expert consultation screening indicators.

Measurement Standard	The first round of consultation			The second round of consultation		
	mean	standard deviation	boundary value	mean	standard deviation	boundary value
Full score frequency (%)	43.889	25.517	18.372	49.521	25.479	24.042
Arithmetic mean	4.135	0.665	3.470	4.354	0.513	3.841
Variation of mean	0.162	0.063	0.225	0.118	0.035	0.153

### Determination of indicator weights

After the screening process of evaluation indicators, the supply evaluation index system for “Internet + public health service” was constructed. It was considered that the impact of each index on the evaluation result is different, and the degree of importance in the entire evaluation index system is also different, it is necessary to assign corresponding weights to each index. According to the scoring and feedback from the experts, each selected index was normalized and its weight was determined reasonably. The final results are shown in Table 5.

**Table 5:** Internet+ public health service supply assessment index system and its weight.

first-level indicators	second-level indicators	third-level indicators	Weight (%)
Internet + Public Health Service Foundation	Information platform for public health services	Proportion of public health service information facilities and equipment meeting standards	4.069
		Proportion of public health service network security level meeting the target	4.082
		Proportion of primary-level medical and health institutions providing Internet Plus public health services	3.315
		Proportion of WeChat Official Accounts Opened by Primary Medical and Health Institutions	3.075
		Proportion of primary medical and health institutions providing remote diagnosis and treatment services	3.321
	Public health service organization construction	Number of registered general practitioners per 10,000 serving population	3.628
		Number of community-level medical and health institutions per 10,000 people	3.373
		The rate at which family doctors sign up	3.311
O2O supply of public health service projects	Online public health services	The standard filing rate of residents’ electronic health records	3.980
		Utilization rate of residents’ electronic health records	4.160
		Internet reporting rates of infectious diseases	4.185
		Internet reporting rate of public health emergencies	4.065
		Report rate of health supervision and management information network	3.788
	Resident Health Services	Preventive vaccination rates	3.936
		Child health management rates	2.847
		Maternal health management early pregnancy enrolment rate	2.741
		Postpartum visit rate of maternal health management	2.920
		Health management rate for the elderly	2.896
		Standard management rate of hypertension patients	3.095
		Standardized management rate of diabetic patients	3.174
		Standard management rate of patients with severe mental disorders	2.539
		Standardized management rate of tuberculosis patients	2.960
		The rate of TCM health management for the elderly	2.830
		Chinese medicine health management rate for children	2.747
Internet + public health service results	Health status of residents	Blood pressure control rate in hypertensive patients	3.896
		Blood glucose control rate in diabetic patients	3.853
		Total incidence of notifiable infectious diseases (1/100,000)	3.765
	Residents’ attitudes	The awareness rate of basic public health services among residents	3.817
		The satisfaction rate of residents’ basic public health services	3.598

## Application of the indicator system

### 1. Collection of empirical data

After establishing the index system, the relevant data was collected from 15 counties and cities in the eastern, central and western regions of China (In accordance with the requirements of the local health administration, the letter A, B…O is used in this study) to conduct an empirical study on the supply evaluation index system for “Internet + public health service”. The data were mainly obtained from the official information of local health and health committees and statistical bureaus as well as public information on application. For indicators that were not counted in some areas, such as the awareness rate of residents’ basic public health services and the satisfaction rate of residents’ basic public health services, the field research on the indicators was carried out, as shown in Table 6 for specific data.

**Table 6:** Variance interpretation table.

Common factor	Eigenvalue	ratio	Cumulative ratio
1	2.444	40.475	40.475
2	1.536	25.602	66.337
3	1.179	19.656	85.992

### 2. Integrated assessment methodology

#### Analytic hierarchy process

Analytic Hierarchy Process (AHP), a comprehensive evaluation method, was proposed by American operations researcher T. L. Saaty in the 1970s [17]. Based on the decision maker’s empirical judgment and problem-solving mindset, the general assessment objectives are determined in accordance with the purpose of the assessment, then the assessment objects are decomposed on a continuous basis according to the general assessment objectives, multiple measurement levels are constructed and the factors at each level are compared with each other to obtain the segmented assessment objectives at each level. And the bottom-level assessment objectives are used as assessment indicators to measure the degree of achievement of the total assessment objectives. The method is highly systematic, practical and concise, which is widely used to solve complex decision-making scenarios with many objectives, criteria, elements and levels. The specific steps of its operation are as follows:

First, the hierarchical structure model of the system is constructed. The goal level, the criterion level, and the solution level are established in order from top to bottom, and factors at the same level play a dominant role over certain factors at the next level.

Second, the judgment matrix for two-comparison is constructed. Once the hierarchical model is constructed, the elemental relationships between the layers are determined.

Third, the relative weights of each factor under a single level are calculated. The weights are calculated as follows:

$$\lambda_{max}=\frac{1}{n}\sum_{i=1}^{n}\frac{(A{w)}_{i}}{w_{i}}$$ (3)

Fourth, matrix consistency is examined. When calculating the ranking weight vector of each factor at a single level, it is usually against common sense to have a judgment result of "A is more important than B, B is more important than C, and C is more important than A", therefore, it is necessary to test the consistency of the judgment matrix based on the consistency index (C.I.) and the average random consistency index (R.I.). The consistency index C.I. and the consistency ratio C.R. are calculated by the following formula:

$$C.I.=\frac{\lambda_{max}-n}{n-1}$$ (4)

$$C.R.=\frac{C.I.}{R.I.}$$ (5)

λ_max_ is the maximum characteristic root and N is the index order.

Fifth, the synthetic weights of each factor to the top level are calculated. The overall rating index is calculated based on the assessment index, and the overall assessment objectives of the target are evaluated, and the superiority and inferiority ratings of the target are determined based on the magnitude of the overall rating index. The composite score index GI is calculated by the formula:

$$GI=\sum_{j=1}^{m}C_{i}•P_{i}$$ (6)

Pi is the measured value of the ith assessment indicator, and m is the number of assessment indicators. If the indicators are all positive indicators, then a higher value of GI indicates a better assessment status.

### TOPSIS

TOPSIS (Technique for Order Preference by Similarity to an Ideal Solution) method, also known as the pros and cons solution distance method, is an effective and common method in the multi-objective decision analysis of limited schemes first proposed by C. L. Hwang and K. Yoon in 1981 [18]. In the TOPSIS method, there are two basic concepts of "ideal solution" and "negative ideal solution". The ideal solution refers to the fact that each attribute value of the scheme is the best of each alternative scheme, which is the optimal scheme in the assumption. Conversely, a negative ideal solution refers to the scenario where each attribute value is the worst of the alternatives, which is the worst and inferior solution in the scenario. The rule of scheme ordering is to compare each alternative with the ideal solution and the negative ideal solution. If one of the alternatives is the closest to the ideal solution, while far away from the negative ideal solution at the same time, then this scheme is the best among the alternatives.

The basic idea of TOPSIS method is to normalize the original data with the same trend and build the corresponding matrix to get the optimal value vector and the worst value vector, that is, the optimal solution and the worst solution in a finite scheme. Then, the distance between each evaluation object or each evaluation index and the optimal solution and the worst solution is calculated respectively according to the formula, so that the closeness of each evaluation object to the optimal solution can be compared, and based on this, the superiority and inferiority of the evaluation object or each evaluation index is ranked.

The formula for calculating the distance D^+^_i_ and D^-^_i_ between the evaluation schemes and the optimal and the worst schemes is as follows:

$$D^{+}i=\sqrt{\sum_{j=1}^{m}({a^{+}}_{ij}-a_{ij}{)}^{2}}$$ (7)

$$D^{‐}i=\sqrt{\sum_{j=1}^{m}{{(a}^{-}}_{ij}-a_{ij}{)}^{2}}$$ (8)

In the formula, D+i and D-i respectively represent the distance between the i-th evaluation object and the optimal and the worst scheme; A_*ij*_ represents the value of a certain evaluation object I in the J-th index.

*C*_i_ is calculated for the approximation degree between the evaluation index objects and the optimal scheme.


$$C_{i}=\frac{D_{i}^{-}}{D_{i}^{+}+D_{i}^{-}}$$ (9)

The value range of C_*i*_ is between 0 and 1. The closer the value is to 1, the closer the evaluation object is to the optimal level. If the value is closer to 0, it means that the evaluation object is closer to the worst level.

### 3. Evaluation result

#### Reliability and validity test

In terms of reliability testing, internal consistency method was adopted in this study. The Cronbach alpha coefficient of the index system reached 0.992, which exceeded the requirement of 0.7, indicating that the internal consistency of the index is good, the measurement result is stable and reliable, and it has practical application value.

In terms of validity test, the appropriateness test of the index system KMO = 0.827, and the spherical test x^2^ = 1346.401, P<0.001 indicate that the potential factors replacing each indicator are mutually independent. Factor analysis can be selected as the validity detection method [19], and the factor analysis of the 6 second-level indicators is carried out. The principal component information obtained is shown in Table 3. According to the standards that the common factors should be as few as possible, eigenvalue should be greater than or equal to 1.0, and cumulative ratio should be greater than 70%, the principal component extraction was extracted [20]. Since the cumulative contribution rate of the first three principal components of the factors with eigenvalues greater than 1 was 85.992%, the first three principal components could be extracted in this study. Rotating by the maximum variance method, the rotated factor loading matrix was obtained [21]. In the evaluation index system constructed in this research, the common factor of the index can explain the total variance of the index of the 85.992%, and each variable basically has enough intensity load on the corresponding factor, indicating that the validity structure is relatively ideal [22]. The factors were classified and named by the research group. The main indicators dominated by "Factor 1" are public health service organization construction and residents’ attitudes, which could be considered that residents’ perception of public health services are mostly dominated by their own contacts with public health service agencies and public health service personnel, so it could be named as residents’ perception factor; "Factor 2" mainly dominates the public health service informatization platform and resident health service, which can be considered as the opportunity brought by health informatization construction for health service. However, due to the low level of the online application of resident health services, most of the service content needs to be completed offline, thereby it can be named as the health service networking factor; The timelier the reporting of infectious diseases, public health emergencies, and health supervision in the field of public health online services, the lower the incidence of infectious diseases. The more effective the protection of the health status of residents, it can be named as a disease prevention and control factor. “Factor 3" mainly dominates the online public health services and the health status of residents. The timelier the reporting of infectious diseases, public health emergencies, and health supervision in the field of public health online services, the lower the incidence of infectious diseases, and the more effective the protection of the health status of residents. Therefore, it can be referred to as the disease prevention and control factor. As can be seen from Tables 7 and 8, component factors 1–3 reflect the service basis of Internet + Public Health Service, the contents of online and offline service supply and the service result. The result of factor analysis is close to the constructed index system structure, so the design of index system has good structural validity on the whole.

**Table 7:** Composition matrix of secondary indicators.

Second-level indicators	Component 1	Component 2	Component 3
Information platform for public health services	0.198	0.930	0.173
Public health service organization construction	0.938	-0.151	0.176
Public health online services	-0.100	0.282	0.850
Resident Health Services	0.548	-0.703	0.250
Health status of residents	-0.262	0.219	-0.735
Residents’ attitudes	0.964	0.187	-0.040

**Table 8:** Comprehensive assessment of the supply status of Internet+ public health services in 15 cities and counties.

index	weight	A	B	C	D	E	F	G	H	I	J	K	L	M	N	O
A																
A1																
A1.1	4.069	1.000	1.000	1.000	1.000	1.000	1.000	1.000	1.000	1.000	1.000	1.000	1.000	1.000	1.000	1.000
A1.2	4.082	1.000	1.000	1.000	1.000	1.000	1.000	1.000	1.000	1.000	1.000	1.000	1.000	1.000	1.000	1.000
A1.3	3.315	1.003	1.003	1.003	1.003	1.003	1.003	1.003	1.003	1.003	0.953	1.003	1.003	1.003	1.003	1.003
A1.4	3.075	1.316	1.014	1.609	1.630	1.901	1.342	0.083	0.435	0.429	0.342	0.785	0.618	0.860	1.280	1.355
A1.5	3.321	1.465	0.488	1.127	0.628	0.976	0.172	1.465	1.172	0.283	1.465	1.176	0.685	1.312	1.264	1.323
A2																
A2.1	3.628	0.765	0.732	0.649	0.704	0.426	1.061	0.834	0.945	0.621	1.112	0.941	0.695	1.668	2.224	1.622
A2.2	3.373	0.685	0.654	0.623	0.685	0.405	0.717	0.654	0.779	0.717	1.994	2.056	1.090	0.997	0.810	2.150
A2.3	3.311	0.887	0.943	0.581	0.321	0.231	0.757	0.565	0.899	1.034	2.228	1.415	1.305	1.229	1.448	1.157
B																
B1																
B1.1	3.98	1.022	0.920	0.900	0.913	0.976	1.010	1.011	1.097	0.965	1.073	1.014	1.028	1.036	1.014	1.019
B1.2	4.16	1.027	0.993	1.040	0.962	1.168	0.865	1.043	1.301	0.865	0.793	1.429	0.473	0.945	1.110	0.988
B1.3	4.185	1.000	1.000	1.000	1.000	1.000	1.000	1.000	1.000	1.000	0.996	1.000	1.000	1.000	1.000	1.000
B1.4	4.065	1.000	1.000	1.000	1.000	1.000	1.000	1.000	1.000	1.000	1.000	1.000	1.000	1.000	1.000	1.000
B1.5	3.788	1.003	1.003	1.003	1.003	1.003	1.003	1.003	1.003	1.003	0.977	1.003	0.990	1.003	1.003	1.003
B2																
B2.1	3.936	1.006	1.008	1.006	1.008	1.007	1.007	1.004	1.007	1.004	0.937	0.998	1.004	1.008	1.000	0.996
B2.2	2.847	1.018	1.009	1.039	1.020	1.002	1.030	1.006	1.001	1.013	0.808	0.987	0.986	1.035	1.039	1.006
B2.3	2.741	1.009	1.005	0.985	0.964	1.020	0.993	1.052	1.029	1.045	0.786	1.003	1.009	1.050	1.024	1.027
B2.4	2.92	1.029	1.021	1.034	1.017	1.029	1.032	1.012	1.031	1.037	0.800	0.982	0.982	1.033	0.960	1.001
B2.5	2.896	0.827	0.786	0.715	0.670	0.682	1.040	0.995	1.270	1.206	1.086	1.327	1.037	1.110	1.114	1.134
B2.6	3.095	0.970	0.921	0.901	0.857	0.891	1.039	0.891	0.995	1.028	1.439	1.169	1.148	1.085	0.707	0.957
B2.7	3.174	0.895	0.915	1.089	0.803	1.089	0.980	0.953	0.991	1.013	1.407	1.075	1.080	1.063	0.691	0.958
B2.8	2.539	0.955	1.027	1.064	0.977	1.065	1.026	1.006	0.992	1.023	1.035	0.790	1.000	1.035	0.988	1.020
B2.9	2.96	0.993	1.016	0.929	1.016	1.016	1.004	1.010	1.013	0.980	1.008	1.016	1.012	0.998	0.992	0.997
B2.10	2.83	1.040	1.035	0.677	0.596	0.555	1.129	1.027	1.107	0.784	1.099	1.286	1.262	1.190	1.101	1.112
B2.11	2.747	1.103	0.915	0.751	0.941	0.781	1.019	1.055	0.976	0.943	1.167	1.115	1.097	1.035	1.059	1.044
C																
C1																
C1.1	3.896	0.825	0.855	1.025	1.019	1.060	0.997	1.226	0.980	1.025	1.096	1.104	1.052	0.885	0.966	0.886
C1.2	3.853	0.871	0.787	1.012	1.038	1.093	1.063	1.150	0.946	1.102	1.130	1.005	1.089	0.848	0.966	0.900
C1.3	3.765	1.219	1.064	1.347	1.057	1.092	1.070	1.230	0.514	0.962	1.086	0.569	0.761	1.304	1.189	0.536
C2																
C2.1	3.817	0.968	0.937	0.841	0.885	0.947	0.832	0.856	0.822	0.838	1.199	1.105	1.118	1.145	1.293	1.215
C2.2	3.598	0.960	0.938	0.977	0.916	0.868	0.927	0.830	0.839	0.845	1.129	1.036	1.076	1.222	1.262	1.176
AHP																
index	——	0.993	0.930	0.966	0.922	0.945	0.967	0.969	0.970	0.923	1.107	1.081	0.980	1.070	1.089	1.084
sort	——	6	13	11	15	12	10	9	8	14	1	4	7	5	2	3
TOPSIS																
di+	——	0.888	1.073	1.145	1.262	1.423	1.075	1.222	1.123	1.258	1.155	0.866	1.081	0.608	0.785	0.520
di-	——	0.967	0.417	0.817	0.391	0.795	0.956	1.086	0.910	0.469	2.255	1.841	1.161	1.734	1.888	1.672
ci	——	0.522	0.280	0.417	0.237	0.358	0.471	0.470	0.448	0.272	0.661	0.680	0.518	0.740	0.706	0.763
排序	——	6	13	11	15	12	8	9	10	14	5	4	7	2	3	1

### Comprehensive assessment results

The data of 15 counties and cities was conducted forward and normalized, and then AHP and TOPSIS methods were used to comprehensively evaluate the Internet + public health service supply. The obtained results are given in Table 4. Through comparison, it can be found that the ranking results obtained by the two evaluation methods are relatively similar, indicating that the index system has a good stability, which can be applied to the comprehensive evaluation of the supply of Internet + public health services supply in various regions, and has practical value.

## Research findings and outlook

The construction of the "Internet + public health service" supply assessment index system is a complex exploration process. The research group carried out analysis from three dimensions: the construction of a public health service information platform and the construction of public health service organizations formed the basis of Internet + public health services; the process of Internet + public health services was measured by the supply status of basic public health service programs online and offline; the results of Internet+ public health services were measured by the health status and attitude of the residents, so as to build a model of "infrastructure—supply process—service results". In the selection process of the index system, a representative and authoritative group of experts was selected for consultation in this study. The high degree of coordination of the expert group’s opinions indicates that the experts have formed a good consensus on the selection of indicators. In terms of indicator application, an empirical study of the constructed indicator system on 15 counties and cities was conducted, and Cronbach’s α = 0.992 and factor analysis are used for reliability and validity tests, and the results showed that this indicator system has good reliability and validity. In the process of validity analysis, each factor variable with the loading of each variable on the corresponding factor (>0.6) was extracted, and the first three extracted common factors were able to explain 85.99% of the variance in the index system, at the same time each variable basically had a sufficiently strong loading on the corresponding factor, indicating that the scale has good structural validity; In terms of the selection of comprehensive evaluation methods, and AHP hierarchical analysis and TOPSIS method were adopted for comprehensive assessment of the Internet+ public health service provision status in 15 counties and cities.The two methods are combined together to confirm each other and complement each other’s advantages. The ranking results obtained are relatively similar, which demonstrates the stability and practicability of the index system, and can objectively measure the current supply situation of Internet + public health services.

Looking ahead, the mobile Internet will become more mature and popular, as well as public health services will continue to increase. As the supply capacity of "Internet + public health services" in primary health care institutions becomes stronger and stronger, the construction of its evaluation index system also needs to keep pace with the times, and continue to innovate and improve with the development of social economy.The group plans to apply the "Internet + public health services" supply assessment index system to more regions for testing, and consider adding indicators such as organizational resilience to withstand the impact of public health emergencies and residents’ digital health literacy, in order to further improve the comprehensiveness and scientificity of the index system.
